# 间变性淋巴瘤激酶抑制剂不良反应管理西南专家建议（2021年版）

**DOI:** 10.3779/j.issn.1009-3419.2021.102.32

**Published:** 2021-12-20

**Authors:** 可 王, 娟 李, 建国 孙, 力 李, 西 张, 建勇 张, 敏 余, 贤伟 叶, 明 张, 瑜 张, 文秀 姚, 媚娟 黄

**Affiliations:** 1 610041 成都，四川大学华西医院呼吸与危重症医学科 Department of Respiratory and Critical Care Medicine, West China Hospital of Sichuan University, Chengdu 610041, China; 2 610041 成都，四川省肿瘤医院肿瘤内科 Department of Oncology, Sichuan Cancer Hospital, Chengdu 610041, China; 3 400037 重庆，陆军军医大学新桥医院肿瘤科 Department of Oncology, Xinqiao Hospital, Army Military Medical University, Chongqing 400037, China; 4 400042 重庆，陆军特色医学中心（大坪医院）呼吸与危重症医学科 Department of Respiratory and Critical Care Medicine, Army Special Medical Center (Daping Hospital), Chongqing 400042, China; 5 610000 成都，成都市第三人民医院肿瘤科 Department of Oncology, Chengdu Third People's Hospital, Chengdu 610000, China; 6 563000 遵义，呼吸与危重症医学科，遵义医科大学附属医院 Department of Respiratory and Critical Care Medicine, Affiliated Hospital of Zunyi Medical University, Zunyi 563000, China; 7 610041 成都，四川大学华西医院胸部肿瘤科 Department of Thoracic Oncology, West China Hospital of Sichuan University, Chengdu 610041, China; 8 550002 贵阳，贵州省人民医院呼吸与危重症医学科 Department of Respiratory and Critical Care Medicine, Guizhou Provincial People's Hospital, Guiyang 550002, China; 9 650118 昆明，云南省肿瘤医院放射治疗科 Department of Radiation Therapy, Yunnan Cancer Hospital, Kunming 650118, China; 10 550002 贵阳，贵州省人民医院肿瘤科 Department of Oncology, Guizhou Provincial People's Hospital, Guiyang 550002, China

**Keywords:** 间变性淋巴瘤激酶抑制剂, 肺肿瘤, 不良反应, 专家建议, Anaplastic lymphoma kinase inhibitors, Lung neoplasms, Adverse reactions, Recommendations

## Abstract

间变性淋巴瘤激酶（anaplastic lymphoma kinase, *ALK*）融合基因作为肿瘤驱动基因，对非小细胞肺癌（non-small cell lung cancer, NSCLC）的发生和发展至关重要，而靶向*ALK*融合基因已成为*ALK*阳性NSCLC患者的主要治疗手段。第一、二代ALK抑制剂（ALK inhibitor, ALKi）克唑替尼、塞瑞替尼、阿来替尼、恩沙替尼已在中国上市并广泛应用。然而，针对ALKi不良反应尚无统一的管理指导规范，在一定程度上降低甚至限制了ALKi的临床使用及患者获益。本文由四川省肿瘤学会肺癌专业委员会及四川省肿瘤性疾病医疗质量控制中心牵头，针对国内已经获批上市的ALKi，参考国内外临床研究和相关文献，并结合专家实践经验，总结出《ALKi不良反应管理专家建议（2021年版）》，以期为临床提供切实可行的ALKi不良反应的管理策略。

## Abstract (en)

Anaplastic lymphoma kinase (*ALK*) fusion gene, as a tumor driver gene, was crucial for the occurrence and development of non-small cell lung cancer (NSCLC). Recently, targeted *ALK* fusion gene has become the main treatment method for *ALK*-positive NSCLC. The first and second generation ALK inhibitors (ALKi), such as crizotinib, ceritinib, alectinib and ensartinib have been approved in China. However, there was no guidance for the management of ALKi adverse reactions. Therefore, this "Recommendations from experts in the management of adverse reactions to ALK inhibitors (2021 version)" has been summarized, led by Lung Cancer Professional Committee of Sichuan Cancer Society and Sichuan Medical Quality Control Center for Tumor Diseases, to provide practical and feasible strategies for clinical ALKi management specification of adverse reactions.

## 1 背景

肺癌作为全球及我国发病率最高的恶性肿瘤，每年的发病率分别为209万例和78.7万例^[1, 2]^，其中非小细胞肺癌（non-small cell lung cancer, NSCLC）约占80%-85%^[3]^。NSCLC的预后较差，5年的总体生存率仅为16%^[4-7]^。近十年来，随着人们对NSCLC发病机制的认识不断深入，间变性淋巴瘤激酶（anaplastic lymphoma kinase, *ALK*）融合基因被发现是NSCLC的第二种常见肿瘤驱动基因，可通过异常激活细胞内信号传导来促进肿瘤细胞的生长，ALK基因重排约占NSCLC的3%-7%，且在年轻的不吸或少烟患者中更为常见^[8]^。由于全球肺癌的总体发病率较高，ALK阳性NSCLC患者的绝对数量仍然是较高的。

ALK抑制剂（ALK inhibitor, ALKi）中，克唑替尼（crizotinib）是第一个被批准用于治疗NSCLC的ALKi，可显著提高患者的无进进展生存期（progression-free survival, PFS）^[9, 10]^，但最终仍会出现耐药进展^[11]^。此后，第二代ALKi塞瑞替尼（ceritinib）^[12, 13]^与阿来替尼（alectinib）^[14, 15]^相继被用于*ALK*阳性NSCLC二线及一线治疗并显示出良好的生存获益。二代ALKi恩沙替尼（ensartinib）二线用于*ALK*阳性NSCLC也有显著获益^[16]^。上述四种ALKi均已在中国上市（恩沙替尼在中国目前仅有二线适应证），其他已在国外上市的ALKi还包括布加替尼（brigatinib）^[17]^、劳拉替尼（lorlatinib）^[18]^。

一般来说，相较于传统的细胞毒性药物，ALKi的总体耐受性良好。但在既往的研究中也发现各类ALKi都存在胃肠道不良反应、药物性肝损伤、心血管不良反应、间质性肺炎（interstitial pneumonia, ILD）或贫血等共有的不良反应^[19-21]^；同时，不同的ALKi也存在着某些特有的不良反应，如视力障碍等^[22]^；某些不良反应虽然少见，但造成的后果却很严重，如心脏不良反应（窦性心动过缓和心肌酶升高等）^[23]^。如何管理好ALKi不良反应，提高患者治疗依从性，提升生活质量与生存获益，已成为临床使用ALKi所面临的一个重要课题^[24-27]^。而目前我国在ALKi所导致不良反应的处理上尚缺乏相关指南或共识的指导，使该类药物的临床应用仍存在潜在的风险，并在一定程度上降低了ALKi的临床应用与患者获益。因此，根据循证证据和临床经验提出的ALKi不良反应相关专家建议具有重要的参考意义，有助于统一规范ALKi的管理。本文由四川省肿瘤学会肺癌专业委员会及四川省肿瘤性疾病医疗质量控制中心牵头，针对国内已经获批上市的4种ALKi，回顾和总结其相关临床研究并参考国内外文献，结合专家的临床实践经验，总结了ALKi不良反应的临床管理建议，以期能为ALKi的临床应用提供指导策略和意见。

## 2 ALKi相关不良反应及其处理

### 2.1 胃肠道不良反应

胃肠道不良反应是ALKi最常见的不良反应，主要包括恶心、呕吐、腹泻、便秘等。

#### 2.1.1 恶心呕吐

##### 2.1.1.1 发生率

恶心呕吐属于ALKi常见的不良反应，总体发生率较高，但多为1级-2级不良反应（表 1）。

**表 1:** 恶心呕吐的发生率 Incidence of nausea and vomiting

ALKi	研究缩写	区域	线数	剂量	恶心			呕吐	
					发生率	≥3级发生率		发生率	≥3级发生率
克唑替尼	Profile 1014^[9]^	全球	一线	250 mg *bid*	56%	1%		46%	2%
	Profile 1029^[24]^	东亚	一线	250 mg *bid*	51%	0		52.9%	0
塞瑞替尼	ASCEND-4^[13]^	全球	一线	750 mg *qd*	69%	3%		66%	5%
	ASCEND-5^[12]^	全球	二线	750 mg *qd*	66%	8%		52%	8%
	ASCEND-8^[25]^	全球	一线	450 mg随餐*qd*	41.7%	0		38.9%	1.9%
				750 mg空腹*qd*	57.3%	5.5%		63.6%	3.6%
阿来替尼	ALEX^[26]^	全球	一线	600 mg *bid*	48%	3%		38%	3%
	ALUR^[27]^	全球	二线	600 mg *bid*	1.4%	0		NG	NG
恩沙替尼	中国Ⅱ期研究NCT03215693^[16]^	中国	二线	225 mg *qd*	10%	0		11%	0
ALKi：间变性淋巴瘤激酶抑制剂									

##### 2.1.1.2 评估与分级

恶心呕吐的分级可以参考美国国立癌症研究所（National Cancer Institute, NCI）发布的常见不良反应术语评定标准（Common Terminology Criteria for Adverse Events, CTCAE）5.0^[28]^（表 2）。

**表 2:** 恶心呕吐的症状 Symptoms of nausea and vomiting

等级	症状	
	恶心	呕吐
1	食欲降低，不伴进食习惯改变	不需要进行干预
2	经口摄食减少不伴明显的体重下降，脱水或营养不良	门诊静脉补液：需要进行医学干预
3	经口摄入能量和水分不足；需要鼻饲，全肠外营养或者住院	需要鼻饲、全胃肠外营养或住院治疗
4	-	危及生命
5	-	死亡

##### 2.1.1.3 处理与预防

建议根据拟行抗肿瘤治疗方案的致吐风险、患者自身的高危因素、既往发生恶心呕吐的严重程度，制定个体化的防治方案。同时，止吐方案的制订还应充分考虑同时使用的非抗肿瘤治疗导致恶心呕吐的风险（如患者合并使用阿片类镇痛药等）。在设计止吐方案时要考虑到实际问题，如处理时的背景（住院患者或门诊患者）、首选给药途径（口服、经皮或肠外）、5-羟色胺3（5-hydroxytryptamine 3, 5-HT3）受体拮抗剂作用持续时间和给药间隔时间、患者对每日给予止吐药物（如糖皮质激素）的耐受性、依从性、顺应性等问题和个体的风险因素^[29]^。

对于1级-2级恶心，推荐维持给药剂量，并调整止吐药治疗方案。

对于1级呕吐，维持给药剂量，并调整止吐药治疗方案；对于2级呕吐，建议停药，直至缓解至≤1级药物不良反应，然后维持药物剂量。对于呕吐持续 > 2级药物不良反应，建议停药直至症状缓解至≤1级药物不良反应，然后降低药物剂量重启治疗。

对于3级-4级恶心和（或）呕吐，建议停药，直至药物不良反应缓解至≤1级，然后降低药物剂量重启治疗^[30-32]^。

#### 2.1.2 腹泻

##### 2.1.2.1 发生率

腹泻是ALKi的常见不良反应，总体发生率较高，在9%-85%，其中以1级-2级不良反应为主（表 3）。

**表 3:** 腹泻的发生率 Incidence of diarrhea

ALKi	研究缩写	区域	线数	剂量	腹泻	
					发生率	≥3级发生率
克唑替尼	Profile 1014^[9]^	全球	一线	250 mg *bid*	61%	2%
	Profile 1029^[24]^	东亚	一线	250 mg *bid*	58.7%	0
塞瑞替尼	ASCEND-4^[13]^	全球	一线	750 mg *qd*	85%	5%
	ASCEND-5^[12]^	全球	二线	750 mg *qd*	72%	4%
	ASCEND-8^[25]^	全球	一线	450 mg随餐*qd*	57.4%	0.9%
				750 mg空腹*qd*	79.1%	9.1%
阿来替尼	ALEX^[26]^	全球	一线	600 mg *bid*	12%	0
	ALUR^[27]^	全球	二线	600 mg *bid*	2.9%	NG
恩沙替尼	中国Ⅱ期研究NCT03215693^[16]^	中国	二线	225 mg *qd*	3.1%	NG

##### 2.1.2.2 临床诊断

腹泻的临床表现主要为大便次数明显增多和大便性状的改变。通常，腹泻时的大便性状可表现为稀便、水样便、黏脓便或脓血便。严重腹泻时，患者可出现口渴、皮肤粘膜弹性变差等脱水症状，少数患者还会伴有明显中毒症状（烦躁、精神萎靡、嗜睡、面色苍白、高热或体温不升、外周白细胞计数明显增高等）的表现^[33]^。

##### 2.1.2.3 评估与分级

腹泻除了影响患者的消化吸收功能和水电解质的平衡，还会影响药物的吸收，宜尽早治疗。然而肿瘤疾病本身也可导致腹泻的发生^[34]^；例如肿瘤患者通常会伴有肠道菌群紊乱^[35]^，而肠道菌群紊乱同样会导致腹泻^[36]^。因此，在诊断时，应注意鉴别非ALKi引起的腹泻。对腹泻的分级标准可采用NCI发布的CTCAE 5.0^[28]^。

除按上述标准进行分级外，还应对下列内容进行评估^[37]^：①确认出现腹泻症状的时间及持续时间；②记录排便次数及排便性状；③评估是否有发烧、晕眩、痉挛等症状，以排除伴随其他更严重副作用的可能；④评估患者的饮食特点与对先前治疗的依从性。

##### 2.1.2.4 处理与预防

对于ALKi患者，可参考表皮生长因子受体酪氨酸激酶抑制剂（epidermal growth factor receptor-tyrosine kinase inhibitor, EGFR-TKI）预防及处理腹泻的方案^[38]^。收集患者接受ALKi治疗开始前6周的排便情况，以便更好地评估ALKi导致腹泻的状况；在治疗开始前收集患者同时服用的其他药物以及其他临床状况，以便评估药物对消化系统潜在的影响，对可能导致消化系统不良反应的药物相互作用也应进行评估；ALKi治疗期间应低脂低纤维饮食，忌食用咖啡因、酒精、奶制品、脂肪、纤维、橘子汁、葡萄汁以及辛辣食物，少食多餐；不得服用泻药，除非有医嘱。对ALKi导致腹泻的患者，处理措施可参考EGFR-TKI导致腹泻的处理方案（表 4、表 5）。

**表 4:** 腹泻的分级 Grade of diarrhea

分级	临床表现
1	与基线相比，大便次数增加每天 < 4次；造瘘口排出物轻度增加
2	与基线相比，大便次数增加每天4次-6次；造瘘口排出物中度增加；借助于工具的日常生活活动受限
3	与基线相比，大便次数增加每天≥7次；需要住院治疗；与基线相比，造瘘口排出物重度增加；自理性日常生活活动受限
4	危及生命；需要紧急治疗
5	死亡

**表 5:** 腹泻的处理 Management of diarrhea

分级	管理	治疗
1级-2级	（1）密切观察，避免脱水；停用软便剂，每天饮用1 L等渗液体； （2）改变饮食（避免摄取乳制品、清淡饮食、少量多餐）； （3）第2级腹泻持续时间超过48 h：评估是否有脱水或电解质失衡的状况，并考虑给予输液，每天饮用1 L-1.5 L等渗液体	（1）使用相同剂量的药物继续治疗；（2）使用洛哌丁胺、益生菌和思密达。洛哌丁胺从4 mg开始（2片），在此之后，每次腹泻后、或每隔4 h服用2 mg（1片）（最高剂量16 mg/d），直到腹泻停止达12 h为止； （3）第2级腹泻持续时间超过48 h将暂停用药，并继续使用洛哌丁胺（最高剂量16 mg/d）、益生菌和思密达治疗，加用可待因（30 mg，*bid*）直到缓解至1级或以下，降低ALKi原剂量，以低剂量重启治疗
3级-4级	（1）让患者住院监测，并采集粪便样本进行显微镜检查； （2）每天饮用1 L-1.5 L等渗液体，积极给予静脉输液补充至少24 h	（1）暂停使用ALKi直到缓解至1级及以下，降低ALKi原剂量，以低剂量重启治疗； （2）使用洛哌丁胺（最高剂量16 mg/d）； （3）益生菌和思密达继续治疗，加用可待因（30 mg, *bid*）；若患者的白细胞及中性粒细胞比例增加，则考虑给予预防性抗生素治疗： ①严重时，可考虑加用生长抑素； ②治疗后腹泻于14天内没有缓解至1级及以下，应给予最佳支持疗法并停用ALKi

#### 2.1.3 便秘

##### 2.1.3.1 发生率

便秘是ALKi的常见不良反应，总体发生率较高，在10.2%-43%，但基本均是1级-2级不良反应（表 6）。

**表 6:** 便秘的发生率 Incidence of constipation

ALKi	研究缩写	区域	线数	剂量	便秘	
					发生率	≥3级发生率
克唑替尼	Profile 1014^[9]^	全球	一线	250 mg *bid*	43%	2%
	Profile 1029^[24]^	东亚	一线	250 mg *bid*	32.7%	0
塞瑞替尼	ASCEND-4^[13]^	全球	一线	750 mg *qd*	19%	0
	ASCEND-5^[12]^	全球	二线	750 mg *qd*	19%	0
	ASCEND-8^[25]^	全球	一线	450 mg随餐*qd*	10.2%	0
				750 mg空腹*qd*	14.5%	0
阿来替尼	ALEX^[26]^	全球	一线	600 mg *bid*	NG	NG
	ALUR^[27]^	全球	二线	600 mg *bid*	18.6%	0
恩沙替尼	中国Ⅱ期研究NCT03215693^[16]^	中国	二线	225 mg *qd*	18%	0

##### 2.1.3.2 临床诊断

便秘表现为排便困难和（或）排便次数减少、粪便干硬。排便困难包括排便费力、排出困难、排便不尽感、肛门直肠堵塞感、排便费时和需辅助排便。其中排便次数减少指每周排便 < 3次^[39]^。

##### 2.1.3.3 评估与分级

便秘的诊断可以参考2016年修订的罗马Ⅳ标准，即自发排便频率 < 3次/周^[39, 40]^。便秘分级可采用NCI发布的CTCAE 5.0^[28]^（表 7）。

**表 7:** 便秘的分级 Grade of constipation

分级	临床表现
1	偶然或间断性出现；偶尔需要使用粪便软化剂、轻泻剂、饮食习惯调整或灌肠
2	持续症状，需要有规律的使用轻泻剂或灌肠；借助于工具的日常生活活动受限
3	需手工疏通的顽固性便秘；自理性日常生活活动受限
4	危及生命；需要紧急治疗
5	死亡

##### 2.1.3.4 处理与预防

增加膳食纤维和水的摄入、增加运动等生活方式调整可以预防便秘。对于临时性的便秘，可使用开塞露缓解。对于轻、中度便秘患者，可以采用容积性泻剂和渗透性泻剂，常用药物包括欧车前、聚卡波非钙和麦麸等以及聚乙二醇和乳果糖。对于特别严重的患者，可以短期、间断使用刺激性泻剂（包括比沙可啶、酚酞、蒽醌类药物和蓖麻油等）作为补救措施以增强肠道动力和刺激肠道分泌^[39]^。

### 2.2 药物相关性肝损伤

药物相关性肝损伤（drug-induced liver injury, DILI）是ALKi比较常见的不良反应，以血清谷丙转氨酶（alanine aminotransferase, ALT）、谷草转氨酶（aspartate transaminase, AST）、碱性磷酸酶（alkaline phosphates, ALP）、谷氨酰转肽酶（glutamyl transpeptidase, GGT）和总胆红素（total bilirubin, TBiL）等实验室指标升高为主要判断依据。ALKi在体内主要通过肝脏细胞色素P3A（cytochrome P3A, CYP3A）酶系代谢^[41]^，推测ALKi导致DILI的原因可能与其活性代谢产物的代谢有关。

#### 2.2.1 发生率

在4种药物的Ⅱ期/Ⅲ期临床研究中均有DILI的报道（表 8）。

**表 8:** DILI的发生率 Incidence of DILI

ALKi	研究缩写	区域	线数	剂量	ALT升高			AST升高			ALP升高			GGT升高			TBIL升高	
					发生率	≥3级发生率		发生率	≥3级发生率		发生率	≥3级发生率		发生率	≥3级发生率		发生率	≥3级发生率
克唑替尼	Profile 1014^[9]^	全球	一线	250 mg *bid*	36%	14%		NG	NG		NG	NG		NG	NG		NG	NG
	Profile 1029^[24]^	东亚	一线	250 mg *bid*	NG	NG		69.2%	11.5%		NG	NG		NG	NG		NG	NG
塞瑞替尼	ASCEND-4^[13]^	全球	一线	750 mg *qd*	60%	31%		53%	17%		29%	7%		37%	29%		NG	NG
	ASCEND-5^[12]^	全球	二线	750 mg *qd*	43%	21%		37%	14%		23%	6%		23%	21%		NG	NG
	ASCEND-8^[25]^	全球	一线	450 mg随餐*qd*	40.7%	17.6%		35.2%	7.4%		18.5%	4.6%		33.3%	22.2%		NG	NG
				750 mg空腹*qd*	40.9%	22.7%		37.3%	10.0%		14.5%	4.5%		23.6%	13.6%		NG	NG
阿来替尼	ALEX^[26]^	全球	一线	600 mg *bid*	15%	5%		14%	5%		NG	NG		1%	1%		15%	2%
	ALUR^[27]^	全球	二线	600 mg *bid*	NG	NG		NG	NG		NG	NG		NG	NG		5.7%	NG
恩沙替尼	中国Ⅱ期研究NCT03215693^[16]^	中国	二线	225 mg *qd*	46%	6%		41%	3%		10%	NG		11%	1%		5.6%	NG

#### 2.2.2 临床表现

DILI的临床表现通常无特异性，患者可有乏力、食欲减退、厌油、肝区胀痛及上腹不适等消化道症状。胆红素升高者可出现皮肤巩膜黄染、大便颜色变浅和皮肤瘙痒等。少数患者可有发热、皮疹、嗜酸性粒细胞增多甚至关节酸痛等过敏表现，还可能伴有其他肝外器官损伤的表现^[42]^。因此，对于治疗过程中出现上述非特异性症状的患者，应考虑到DILI的可能性。但无症状患者，也并不能排除DILI的可能性。

#### 2.2.3 评估与分级

DILI除了非特异性的临床表现与肝酶升高以外，其他影像学检查如超声、计算机断层扫描（computed tomography, CT）、磁共振成像（magnetic resonance imaging, MRI）等对诊断DILI也有临床提示作用。同时，在临床诊断过程中，需要排除肿瘤自身原因与其他非ALKi引起的肝脏损害。DILI的诊断流程与策略以及DILI的肝损伤评估标准（RUCAM量表和肝损伤分级标准），可参考中华医学会药物性肝病学组发布的《药物性肝损伤诊治指南》^[42]^，根据严重程度可以将DILI分为1级-5级（表 9）。

**表 9:** DILI的分级 Grade of DILI

分级	临床表现
1级（轻度肝损伤）	血清ALT和/或ALP呈现可恢复性升高，TBil < 2.5×ULN（2.5 mg/dL或42.75 *μ*mol/L），同时INR < 1.5。大多数患者可以适应，可伴随或不伴随乏力、虚弱、恶心、厌食、右上腹痛、黄疸、瘙痒、皮疹或体重减轻等症状
2级（中度肝损伤）	血清ALT和（或）ALP升高，TBil≥2.5×ULN，或者虽无TBil升高但INR≥1.5。上述症状可能会有加重
3级（重度肝损伤）	血清ALT和（或）ALP升高，TBil≥5×ULN（5 mg/dL或85.5 *μ*mol/L），伴或者不伴有INR≥1.5。患者的症状进一步加重，一般需要入院接受治疗，或者住院的时间延长
4级（急性肝衰竭）	血清ALT和（或）ALP升高，TBil≥10×ULN（10 mg/dL或171 *μ*mol/L），或者每天上升≥1.0 mg/dL（17.1 *μ*mol/L），INR≥2.0或PTA < 40%，可能同时出现腹水或肝性脑病，或与DILI相关的其他器官功能衰竭
5级（致命）	DILI引起的死亡，或需要接受肝移植才能存活
ALT：血清谷丙转氨酶；ALP：碱性磷酸酶；TBil：总胆红素；ULN：上限；INR：国际标准化比值	

#### 2.2.4 处理与预防

由于3级-4级的肝酶升高的总体发生率在30%以下，真正进展为重度DILI的情况相对较少，因此在多数情况下，肝酶升高并非是立即停药的指征。但当出现TBIL升高时，需要引起警惕，若继续用药则有可能诱发肝功能衰竭的危险^[42]^。对于具体的诊断、分级以及治疗，可以参考中华医学会药物性肝病学组发布的《药物性肝损伤诊治指南》^[42]^（表 10）。不同ALKi药物的肝损伤不良反应剂量调整也可参考药物说明书推荐，如塞瑞替尼建议：当ALT或AST > 5×ULN、总胆红素 < 2倍正常值时，应当停止用药，直到ALT/AST恢复到基线或者 < 3倍正常值时，由150 mg剂量慢慢恢复到正常量。当ALT或AST > 3×ULN、总胆红素 > 2×ULN（不存在胆汁淤积或溶血）时，应终止用药。

**表 10:** 肝损伤的治疗措施 Treatment of liver injury

治疗措施	治疗详情
停药	（1）及时停用可疑的肝损伤药物是最为重要的治疗措施，尽量避免再次使用可疑或同类药物； （2）临床上停药可以参考美国食品药品监督管理局（Food and Drug Administration, FDA）在药物临床试验中建议的停药标准。出现下列情况之一建议应考虑停药： ①血清ALT或AST > 8×ULN； ②ALT或AST > 5×ULN，持续2周； ③ALT或AST > 3×ULN，且TBil > 2×ULN或INR > 1.5； ④ALT或AST > 3×ULN，伴逐渐加重的疲劳、恶心、呕吐、右上腹疼痛或压痛、发热、皮疹和（或）嗜酸性粒细胞增多（> 5%）
药物治疗	（1）重型成人患者可选用N-乙酰半胱氨酸（N-acetyl-L-cysteine, NAC），临床越早应用效果越好。成人一般用法：50 mg/kg/d-150 mg/kg/d，总疗程不低于3天； （2）糖皮质激素应仅限用于超敏或自身免疫反应征象明显且停用ALKi后生化指标改善不明显甚或继续恶化的患者，并应充分权衡治疗收益和可能的不良反应； （3）异甘草酸镁可用于治疗ALT明显升高的急性肝细胞型或混合型肝损伤； （4）有经验表明，轻-中度肝细胞损伤型和混合型DILI，炎症较重者可试用双环醇和甘草酸制剂；炎症较轻者可试用水飞蓟素。胆汁淤积型DILI可选用熊去氧胆酸（ursodeoxycholic acid, UDCA）。有报道腺苷蛋氨酸（S-adenosylmethionine, SAMe）治疗胆汁淤积型DILI有效。上述药物的确切疗效有待严格的前瞻性随机对照研究加以证实
肝移植	对出现肝性脑病和严重凝血功能障碍的急性/亚急性肝衰竭以及失代偿性肝硬化，可以考虑肝移植

此外，对于合并病毒性肝炎的患者，如携带乙型肝炎病毒（hepatitis B virus, HBV）或丙型肝炎病毒（hepatitis C virus, HCV）有研究报道TKIs可诱导HBV的活化，其确切机制目前尚不清楚，可能与TKIs的脱靶效应导致HBV的活化有关，因此对于接受TKIs治疗的NSCLC患者，在治疗期间存在有临床意义的HBV活化的风险^[43, 44]^。

目前，对于接受TKIs治疗的HBsAg阳性抗HBS Ag阴性/抗HBC阳性的患者，在开始TKIs治疗前应进行HBV DNA水平的检测。对于HBV DNA水平较高的患者，可以考虑使用抗病毒的预防性治疗。对于已知HBV感染的患者，建议每4周定期进行肝功能检测（包括AST、ALT），并在TKIs治疗前和治疗后的每3个月或当出现肝功能异常时，进行HBV病毒载量水平的监测，一旦HBV DNA水平升高，应考虑给予抗病毒治疗^[43, 44]^。

### 2.3 水肿

水肿是比较常见的不良反应。两项Ⅲ期临床研究^[9, 24]^中克唑替尼水肿的发生率分别为49%与27.9%。两项Ⅱ期研究^[26, 45]^汇总以及Ⅲ期ALEX研究中，阿来替尼所致的外周水肿的发生率分别为28.4%及17%。在中国的Ⅱ期研究中，恩沙替尼所致的面部水肿和外周水肿发生率分别为16%和9%^[16]^。对于水肿的处理，首先应排除其他因素引起的水肿，如心源性、其他药物、低蛋白血症、甲状腺功能减退、肢体血管栓塞等因素导致的水肿。水肿可以通过利尿剂对症处理、抬高下肢、低盐清淡饮食缓解水肿症状，必要时减量甚至暂时停药，密切观察。

### 2.4 皮疹

#### 2.4.1 发生率

皮疹不良反应包括斑丘疹、痤疮样皮炎、红斑、丘疹样皮疹、瘙痒性皮疹和斑状皮疹等表现。在克唑替尼PROFILE 1014 Ⅲ期研究中，皮疹的发生率约为11%^[9]^。在塞瑞替尼ASCEND-5研究中，皮疹的发生率约为13%，且均为轻症^[12]^。阿来替尼在Ⅱ期临床试验（NP28761, NP28673）和Ⅲ期临床试验（ALEX）中接受阿来替尼治疗的患者的皮疹发生率为20%，其中重症为0.7%^[26, 45]^。此外在Ⅲ期ALEX研究中，有5%的光敏反应发生，其中3级-4级为1%^[26]^。在中国的Ⅱ期研究中，恩沙替尼所致的皮疹发生率甚至高达56%，其中3级-4级为6%^[16]^。

#### 2.4.2 处理与预防

大多数ALKi所致的皮疹不良反应样是可防、可控、可逆的^[46-48]^。对轻-中度不良反应，可采取综合防治措施，如生活方式调整及药物干预等措施，提高患者的生活质量，而无需调整靶向药物的剂量或中断治疗从而影响抗癌效果；对于重度不良反应，应系统用药改善症状，必要时减量或暂停抗肿瘤靶向治疗，待皮疹改善后考虑是否调整抗癌方案（表 11）。

**表 11:** 皮疹的治疗措施 Treatment for rashes

表现	分级	处理
痤疮样皮疹	1级	可使用1%-2%的红霉素^[46]^
	2级	可口服土霉素500 mg，*bid*^[47]^
	3级	皮肤科治疗
瘙痒	一般	可使用的抗组胺药包括：左西替利嗪5 mg *qid*、地氯雷他定5 mg *qid*、苯海拉明25 mg-50 mg *tid*、羟嗪25 mg *tid*或非索非那定60 mg *tid*^[47, 48]^
	重度	可使用γ-氨基丁酸受体激动剂（如果患者有肾功能损害则调整剂量），如加巴喷丁每8小时300 mg或普瑞巴林每8小时50 mg-75 mg；三环类药物，如多西平每8小时25 mg-50 mg、阿瑞匹坦3次：第1天125 mg，第2天和第3天各80 mg^[47, 48]^
	严重	皮肤科治疗

### 2.5 贫血

贫血主要指肿瘤患者在疾病进展和治疗过程中发生的贫血，特征表现为外周血中单位容积内红细胞数减少、血红蛋白浓度降低或红细胞比容（hematocrit, HCT）降低至正常水平以下^[49]^。

#### 2.5.1 发生率

贫血是相对比较常见的不良反应，但多以轻症为主。塞瑞替尼在ASCEND-4研究中，贫血的总体发生率为15%，其中3级-4级为2%。阿来替尼的贫血发生率为25%，其中3级-4级为5%^[26]^（表 12）。

**表 12:** 贫血的发生率 Incidence of anemia

ALKi	研究缩写	区域	线数	剂量	发生率	
					发生率	≥3级发生率
克唑替尼	Profile 1014^[9]^	全球	一线	250 mg *bid*	9%	0
	Profile 1029^[24]^	东亚	一线	250 mg *bid*	21.2%	2.9%
塞瑞替尼	ASCEND-4^[13]^	全球	一线	750 mg *qd*	15%	2%
	ASCEND-5^[12]^	全球	二线	750 mg *qd*	NG	NG
	ASCEND-8^[25]^	全球	一线	450 mg随餐*qd*	NG	NG
				750 mg空腹*qd*	NG	NG
阿来替尼	ALEX^[26]^	全球	一线	600 mg *bid*	25%	5
	ALUR^[27]^	全球	二线	600 mg *bid*	NG	NG
恩沙替尼	中国Ⅱ期研究NCT03215693^[16]^	中国	二线	225 mg *qd*	9%	0

#### 2.5.2 临床表现

外周血血红蛋白低于正常成人外周血血红蛋白的范围标准（成年男性：120 g/L-160 g/L、成年女性：110 g/L-150 g/L）^[50]^。

#### 2.5.3 评估与分级

贫血可能由许多原因导致，其中部分原因可能与肿瘤无直接关系。因此，评估的总目标是找出贫血的特点并在初始治疗前确定任何潜在的可纠正的基础性合并症。贫血的分级标准可参考《中国肿瘤化疗相关贫血诊治专家共识》中关于贫血的分级^[49]^：①0级（正常）： > 正常值下限；②1级（轻度）：90 g/L-正常值下限；③2级（中度）：60 g/L-90 g/L；④3级（重度）：30 g/L-60 g/L；⑤4级（极重度）： < 30 g/L（正常值低限：男性120 g/L；女性110 g/L）。

#### 2.5.4 处理与预防

贫血的治疗方法主要包括输血治疗、促红细胞生成（erythropoietin, EPO）治疗和补充铁剂等。具体的处理措施可参考《中国肿瘤化疗相关贫血诊治专家共识》中关于贫血的具体治疗措施^[49]^（表 13）。

**表 13:** 贫血的治疗措施 Treatment of anemia

治疗措施	治疗详情
输血治疗	血红蛋白 < 60 g/L若： ①无症状，无明显的合并疾病：定期再评估； ②无症状，但有合并疾病（心脏病包括输血相关循环超负荷和冠心病、肺源性心脏病、脑血管疾病）或高风险（近期化疗或放疗伴有血红蛋白快速下降）：考虑红细胞输注； ③有症状（持续心动过速、呼吸急速、胸痛、运动性呼吸困难、轻度头痛、晕厥、影响工作和惯常活动的中度疲劳）：进行红细胞输注
促红细胞生成治疗	EPO 150 U/kg或10 kU每周3次，或36 kU每周1次，皮下注射，1个疗程4周-6周。任何情况下血红蛋白≥120 g/L，则停止使用EPO
铁剂补充	口服铁剂：优点：使用方便。缺点：服用后仅约10%被人体吸收，同时胃肠道刺激症状比较严重。部分患者对口服铁剂过敏。口服铁剂包括硫酸亚铁（常用）、富马酸亚铁（常用）、葡萄糖酸亚铁、琥珀酸亚铁和乳酸亚铁 肠道外铁剂：优点：能够被人体完全吸收，起效快，无胃肠道刺激症状。缺点：需要注射使用。肠道外铁剂包括右旋糖酐铁、葡萄糖酸铁、蔗糖铁。考虑到患者耐受性和药代动力学的原因，推荐使用蔗糖铁，用于对口服铁不耐受或无反应的患者缺铁治疗，也可用于接受EPO类药物治疗的肿瘤患者 右旋糖酐铁：需要先应用试验剂量，尤其是既往有其他药物过敏的患者。推荐的右旋糖酐铁为低分子右旋糖酐铁
EPO：促红细胞生成素	

### 2.6 肌肉骨骼痛及结缔组织不良反应

在阿来替尼的三项临床研究（NP28761、NP28673、ALEX）中，28%的患者报告了肌痛，包括肌痛事件（22%）和肌肉骨骼疼痛（7.4%），其中多数事件为1级-2级，3级发生率为0.7%。在362例具有肌酸磷酸激酶（creatine phosphokinase, CPK）实验室数据的患者中，43%的患者出现CPK升高，3级发生率为3.7%^[26, 45]^。

肌肉骨骼痛时多伴有CPK升高，当CPK升高 > 5×ULN，可以暂停药物治疗，直到恢复至基线水平或者≤2.5×ULN，然后以暂停前的剂量恢复给药；当CPK升高 > 10×ULN或者第二次发生CPK升高 > 5×ULN，建议暂停治疗，直到恢复至基线水平或者≤2.5×ULN后，降低药物剂量重启治疗。

### 2.7 肾脏毒性

肾脏毒性临床多表现为血肌酐升高，在塞瑞替尼ASCEND-4和ASCEND-8研究中，血肌酐升高的发生比例分别为22%与21.3%，其中3级-4级为2%与0%^[13, 25]^。除此之外，有报道使用克唑替尼的患者发生肾囊肿。发生肾囊肿的原因尚不完全清楚，但有报道发现，停止克唑替尼治疗后肾囊肿出现消退^[51]^。此外，对于肾功能不全的患者，轻度/中度患者，无需调整剂量。对于重度肾功能损害者的处理，目前尚缺乏相关研究，建议先中断治疗待肾功能改善后，以较低剂量重新开始治疗^[52]^。

### 2.8 心脏不良反应

ALKi引起的心脏不良反应主要表现为QT间期延长、心动过缓等。

#### 2.8.1 发生率

心脏不良反应在4种ALKi中均有报道，但总体发生率较低。回顾性分析报道显示，在1, 167例使用克唑替尼的患者中有1.4%的患者出现QT间期≥500 ms；1, 136例患者中有4.4%的患者的QT间期较基线延长≥60 ms^[53]^。克唑替尼患者心动过缓的总体发生率 < 2%，与基线相比平均心率减少了26.1次/min，有69%的患者至少发生过一次窦性心动过缓（≤60次/min）^[54]^。在塞瑞替尼的ASCEND-2研究中，有7.9%的患者出现QT间期延长，其中0.7%出现了3级QT间期延长^[55]^；在ASCEND-5研究中，QT间期延长的发生率为11%，其中3级/4级为1%^[12]^；其心动过缓的发生率 < 2%^[41]^。在阿来替尼患者中，QT间期延长的发生率为3%，其中3级-4级为2%；心动过缓的发生率1%，且均为轻度^[14]^。在中国的Ⅱ期研究中，并未观察到恩沙替尼导致的QT间期延长；引起任意级别心动过缓的发生率为1.9%^[16]^。

#### 2.8.2 临床表现

QT间期延长的临床表现可有头晕、心悸、晕厥、癫痫或不明原因的意识丧失等。心动过缓（< 60次/min）可导致疲劳、头晕、晕厥、胸闷、心悸、低血压和疲劳，严重者可以发生晕厥、低血压等血流动力学障碍的表现。虽然心脏不良反应的临床发生率不高，但容易被临床忽视而造成严重后果。目前，对于药物引起的QT间期延长和心动过缓所造成的远期影响尚不完全清楚。

#### 2.8.3 评估与分级

尽管ALKi引起的心脏不良反应的发生率较低，一旦发生，容易造成严重后果，所以应时刻提高警惕，早期发现心脏异常。一旦出现头晕、胸闷、心悸等临床症状，应尽快完成炎症指标（如血沉和C反应蛋白）、心肌酶、前脑利钠肽、心电图、超声心动图等检测评估，应注意动态观察上述检测指标以评估病情严重程度，同时进行病因的鉴别诊断。对于心脏不良反应的分级可以参考CTCAE 5.0^[28]^（表 14）。

**表 14:** 心脏不良反应的分级标准 Grade of adverse cardiac reactions

分级	临床/实验室表现	
	QT间期延长	窦性心动过缓
1	QT间期450 ms-480 ms	无症状，无需治疗
2	QT间期481 ms-500 ms	有症状，无干预指征；开始改变药物治疗
3	平均QT间期≥501 ms；比基线期 > 60 ms	有症状，需要治疗
4	尖端扭转型室速；阵发性室性心动过速；严重心律不齐体征/症状	危及生命；需要紧急治疗
5	-	死亡

#### 2.8.4 处理与预防

某些患者可能同时患有心脏病或高血压，在使用ALKi之前需仔细评估ALKi治疗的可能性，尽可能避免与具有延长QT间期风险的药物联用。对于有QT间期延长或心动过缓病史的患者，应谨慎使用ALKi，并定期监测心电图。此外，当服药患者存在呕吐、腹泻或肾功能损害时，应注意因电解质紊乱可能会增加QT间期延长的风险，并定期监测电解质。有心脏病史的患者和正在服用可能延长QT间期药物的患者应考虑使用心电监护^[41]^（表 15）。

**表 15:** 心脏不良反应的治疗措施 Treatment of adverse cardiac reactions

分级	处理^[30-32]^
1级	无需处理
2级	先评估是否存在已知导致心动过缓的联合用药以及降压药物，如果发现了导致心动过缓的联合用药应调整药物剂量或停用该药物，则重新开始使用此前剂量的ALKi治疗，直至恢复至无症状的心动过缓或者心率≥60 bpm；如果没有发现导致心动过缓的联合用药，或者如果不能停用或者调整导致心动过缓的联合用药剂量，则ALKi减量，直至恢复至无症状心动过缓或者心率≥60 bpm
3级	应停药，直至恢复至无症状的心动过缓或者心率≥60 bpm，如果可以调整或者停用联合用药，则建议减少ALKi剂量重新开始治疗，定期监测
4级	永久停药

### 2.9 ILD

ILD是以肺间质为主要病变的众多异质性疾病的总称，以局灶或弥漫性肺间质的非感染性炎性改变和进行性纤维化，甚至发展为呼吸衰竭和心功能不全为病变特点^[56]^。ALKi导致的ILD虽然发生率较低，但一旦发生后果可能非常严重^[57]^。

#### 2.9.1 发生率

克唑替尼ILD的发生率在1%-2%之间。PROFILE1014研究中，ILD的发生率为1%并导致患者永久停药^[9]^。在PROFILE1029研究中，也发生了1例重度ILD^[24]^。在塞瑞替尼治疗的患者中有1.0%-4.0%出现了肺炎或ILD，3%的患者出现3级-4级ILD^[55, 58]^。在253例阿来替尼临床研究患者中，仅有1例（0.4%）出现3级ILD^[59]^。在中国的Ⅱ期研究中，并未观察到恩沙替尼导致的ILD^[16]^。

#### 2.9.2 临床表现

ILD主要表现为咳嗽，以干咳为主；渐进性呼吸困难，活动后呼吸困难更明显；发热；全身伴随症状，如食欲减退、消瘦、乏力等；最终可导致严重的双肺纤维化和低氧血症，严重者可引起呼吸衰竭。

#### 2.9.3 评估与分级

临床上ILD的症状很有可能是疾病本身而非药物引起，如疾病进展、继发感染或既往胸部放疗、基础肺间质纤维化改变等引起的。因此，需要在确认排除上述问题之后再行药物的减量或停药，可通过实验室检测（血常规、血培养、痰培养等）以及影像学检测等明确。ILD的临床分级可参考美国国家综合癌症网（National Comprehensive Cancer Network, NCCN）指南以临床表现结合影像学进行分级^[60]^（表 16）。

**表 16:** ILD的分级标准 Grade of ILD

分级	症状	活动能力	影像学改变	治疗干预
1	无症状；仅临床检查发现	正常	< 25%	无需干预
2	有症状	工具性日常生活活动受限	25%-50%	需要药物治疗
3	症状严重	个人日常生活自理活动受限	51%-75%	需氧疗
4	危及生命的呼吸功能衰竭	卧床	> 75%	紧急抢救（如气管插管或气管切开）
5	死亡			

#### 2.9.4 处理与预防

ILD的治疗目标是抑制炎症反应，促进渗出吸收，防止肺间质纤维化，保护心肺功能。主要治疗方法包括氧疗、机械通气、糖皮质激素以及按需进行经验性抗感染治疗，密切监测病情变化，及时进行再评估和检查。

具体处理措施可以参考EGFR-TKI相关性ILD的处理措施^[48]^：（1）临床上一旦发生或怀疑ILD时，应立即停止ALKi；若有引起或加重ILD的合并用药（如博来霉素、胺碘酮等），可换用其他对ILD无影响的药物；（2）对于确诊或高度怀疑ALKi引起的ILD，应立即开始以糖皮质激素治疗，并注意补充钙及维生素D，监测血糖，预防消化道出血：①1级：密切监测症状体征和血液学检查，一旦恶化，按2级-4级治疗；②2级：起始甲基强的松龙0.5 mg/kg/d-1.0 mg/kg/d或等效药物，持续2周-4周症状体征恢复后缓慢减量，总疗程至少6周；③3级：起始甲基强的松龙1.0 mg/kg/d-2.0 mg/kg/d或等效药物，持续2周-4周症状体征恢复后缓慢减量，总疗程至少8周；④4级：甲基强的松龙500 mg/d-1, 000 mg/d冲击治疗，3天后甲基强的松龙1 mg/kg/d-2 mg/kg/d，持续2周-4周症状体征恢复后缓慢减量，总疗程至少8周-10周；（3）经验性抗生素抗感染治疗（按需或根据微生物学检查结果选择敏感抗感染药物）；（4）氧疗：推荐参照慢性阻塞性肺疾病氧疗指征，静息状态低氧血症[动脉血氧分压（arterial partial pressure of oxygen, PaO_2_）≤55 mmHg或血氧饱和度（blood oxygen saturation, SaO_2_）≤88%]的ILD患者接受长程氧疗，氧疗时间 > 15 h/d；（5）发生呼吸衰竭时行机械辅助通气。

### 2.10 视觉障碍

#### 2.10.1 发生率

视力障碍是ALKi特有的不良反应。在克唑替尼的2项Ⅲ期研究中，其总体发生率在60%-71%，其中3级-4级为0-1%^[9, 61]^，视力障碍主要表现为视觉减退、视力模糊、飞蚊症、畏光和复视^[61, 62]^。阿来替尼也有报道3%患者存在轻度视觉不良反应^[26]^。

#### 2.10.2 处理与预防

一般来说，视觉障碍多表现为短暂性的（< 30 s或30 s-60 s），且会随着靶向药物使用时间的增加而减少，对患者的日常生活影响较小或没有影响，因此一般不需要特殊的处理^[53]^。如果视觉障碍持续存在或严重程度恶化，也应考虑视功能和/或神经学评估，以排除非药源性的视网膜病变、视神经病变或中枢神经系统的病变，必要时眼科专科就诊。

### 2.11 代谢失衡

代谢失衡主要表现为低蛋白血症、低钙、低磷、高糖等。在克唑替尼患者中，低蛋白血症与低钙血症的发生率分别为11%和10%^[24]^。在ASCEND-8研究中，使用塞瑞替尼450mg *qd*的患者中，高糖血症的发生率为12.0%，其中3级-4级不良反应占7.4%。对于患者报告重度口渴或排尿频次增加或饥饿感增加、头痛、思考困难或专注力障碍等，应注意监测电解质和血糖，并给予对症处理，反复代谢失衡者应考虑减量甚至暂时停药。

### 2.12 超敏反应

有研究报道曾有2例使用克唑替尼的患者发生了超敏反应，表现为服药后数小时出现全身皮疹。这些患者接受了脱敏治疗：首先给予单剂氯雷他定（10 mg）、西替利嗪（10 mg）和非索非那定（180 mg），1 h后，每15 min给予1次克唑替尼，剂量递增，持续3 h。脱敏后，患者继续服用克唑替尼未再出现超敏反应的症状^[63]^。

## 3 其他ALKi及不良反应

布加替尼是一种可逆的EGFR/ALK双重抑制剂，属于第二代ALKi。在该药的随机、多中心Ⅱ期试验中，纳入了222例既往接受过克唑替尼治疗的*ALK*阳性NSCLC患者，结果表明使用布加替尼作为克唑替尼耐药后的治疗方案，尤其是对脑转移的控制和治疗，具有明显的效果。两个剂量组常见的急性不良事件（adverse event, AE）为恶心（33%和40%）、腹泻（19%和38%）、头痛（28%和27%）和咳嗽（18%和34%），主要为1级-2级；219例接受治疗的患者中有14例发生了一部分早期发作的肺部不良事件^[17]^。基于该研究结果，布加替尼于2017年获FDA批准为*ALK*阳性NSCLC患者二线治疗（2020年已扩展适应证为一线）。对于1级或2级AE，应中断然后调整降低剂量，对于复发或较高级事件应停止给药。对于罕见的早期肺部事件，如中断剂量可以30 mg开始给药，然后以每3天30 mg递增至可耐受的全剂量^[64]^。

劳拉替尼是一种高效的第三代ALKi，属于ALK/ROS1双靶点抑制剂，其设计特点是可穿透血脑屏障。该药的Ⅱ期临床试验纳入了275例*ALK/ROS1*阳性晚期NSCLC患者，其中大多为具有脑转移并且进行过ALK类药物治疗甚至是化疗的患者，结果显示：一线直接使用劳拉替尼治疗的患者有效率达90%，作为二线或三线药物使用有效率依然高达69%；所有患者中最常见的AE为高胆固醇血症（81%）和高甘油三酯血症（60%）^[65]^。2018年11月，FDA基于劳拉替尼的Ⅰ期/Ⅱ期研究结果，批准其上市用于*ALK*阳性转移性NSCLC患者的二线治疗；2019年5月，劳拉替尼也在欧盟获批上市；2021年3月，FDA基于劳拉替尼的Ⅲ期CROWN研究批准了劳拉替尼用于ALK阳性NSCLC患者的一线治疗^[66]^（具体适应证见表 17）。总体上该药耐受性良好，高脂血症、中枢神经系统反应、体重增加、水肿和周围神经病变是其最常见的不良反应。AE的严重程度通常为轻至中度，很少导致永久停药，一般可通过剂量调整和/或标准药物对症治疗来控制^[67]^。

**表 17:** 目前国内外已上市的ALKi种类及常见不良反应 Types and common adverse reactions of ALKi that have been marketed at home and abroad

ALKi	中文名称	英文名称	FDA批准适应证	常见不良反应
第一代	克唑替尼^[53]^	Crizotinib	治疗转移性*ALK*阳性和*ROS1*阳性NSCLC（二线，一线）	视觉障碍、恶心、腹泻、呕吐、便秘、水肿、ALT和AST升高以及疲劳等
第二代	塞瑞替尼^[68]^	Ceritinib	治疗*ALK*阳性NSCLC患者（二线，一线）	腹泻、恶心、ALT升高、AST升高、呕吐、血糖升高和脂肪酶升高等
	阿来替尼^[14]^	Alectinib	晚期/转移性*ALK*阳性NSCLC患者（二线，一线）	疲劳、便秘、水肿、肌痛、贫血、AST升高、ALP升高、CPK升高、高胆红素血症、高血糖症、ALT升高和低血钙症等
	布加替尼^[69]^	Brigatinib	治疗*ALK*阳性NSCLC患者（二线，一线）	恶心、疲劳、腹泻、头痛、咳嗽、脂肪酶升高、高血压等
	恩沙替尼^[16]^	Ensartinib	仅在中国获批治疗*ALK*阳性NSCLC患者（二线）	皮疹、ALT升高、AST升高、肌酐升高、瘙痒、便秘等
第三代	劳拉替尼^[67]^	Lorlatinib	治疗*ALK*阳性NSCLC患者（二线，一线）	高脂血症、中枢神经系统反应（如认知障碍、焦虑、抑郁、语速变慢等）、体重增加、水肿和周围神经病变等
CPK：肌酸磷酸激酶				

目前市场上可获得的ALKi已经发展至第三代产品，布加替尼和劳拉替尼现尚未在中国上市。不同的ALKi所带来的药物不良反应也不尽相同（表 17），因此在临床处理上仍应谨慎。

## 4 总结

一系列的临床研究证明，采用包括克唑替尼、塞瑞替尼、阿来替尼在内的ALKi治疗*ALK*阳性NSCLC疗效确切。NCCN等指南基于这些研究证据，将阿来替尼、布加替尼和劳拉替尼作为*ALK*阳性转移性NSCLC患者的首选一线治疗选择（1类推荐），同时塞瑞替尼也被推荐为具有ALK基因重排的NSCLC患者的一线治疗（1类推荐），克唑替尼仅在特定情况下才被推荐。从安全性角度看，此类药物的临床研究和实践中所报告的多数不良反应均属于轻症，可控可逆。对于部分少见但后果严重的不良反应，早期识别并采取积极的干预措施则是管理不良反应的关键^[68, 69]^。然而目前国内外，尚无针对ALKi不良反应的管理指导规范，因此基于循证证据和临床经验的管理策略的探讨具有重要意义。本文中总结了四种国内上市的ALKi药物临床研究数据、相关文献以及专家的临床实践经验，对于如何管理ALKi的不良反应提出了意见和建议，供临床医师参考，使药物所致的不良反应最小化，避免不必要的减量或停药，提高患者的生存获益和生活质量。当然，由于塞瑞替尼，阿来替尼与恩沙替尼的上市时间还不长，有关不良反应的管理措施还有待药物在真实世界中的应用观察和证据积累。今后还会有其他ALKi新药陆续上市，我们将据此对本建议进行更新和补充。
